# Effectiveness of binocularity-stimulating treatment in children with residual amblyopia following occlusion

**DOI:** 10.1186/s12886-018-0922-z

**Published:** 2018-09-20

**Authors:** Haeng-Jin Lee, Seong-Joon Kim

**Affiliations:** 1 Department of Ophthalmology, Seoul National University College of Medicine, Seoul, South Korea; 2 Seoul Artificial Eye Center, Seoul National University Hospital Clinical Research Institute, Seoul, South Korea; 3 Department of Ophthalmology, Seoul National University Hospital, 101 Daehak-Ro, Jongno-Gu, Seoul, 110-744 Republic of Korea

**Keywords:** Amblyopia, Residual amblyopia, Binocularity-stimulating treatment, Binocular treatment

## Abstract

**Background:**

To evaluate the effectiveness of binocularity-stimulating treatment in children with residual amblyopia following occlusion therapy for more than 6 months.

**Methods:**

Of patients with amblyopia caused by anisometropia and/or strabismus, patients with residual amblyopia following more than 6 months of occlusion therapy were included. Subjects underwent one of the following types of binocularity-stimulating therapy: Bangerter foil (BF), head-mounted display (HMD) game, or BF/HMD combination (BF + HMD). Factors including age, sex, types of amblyopia, visual acuity, and duration of treatment were investigated. Baseline and final (after at least 2 months of treatment) visual acuity were also compared.

**Results:**

Twenty-two patients with a mean age of 8.7 ± 1.3 years were included. Seven patients had anisometropic amblyopia, 8 patients had strabismic amblyopia, and 7 patients had combined amblyopia. After 4.4 ± 1.8 months of treatment, logarithm of the minimum angle of resolution (logMAR) visual acuity in the amblyopic eye improved from 0.22 ± 0.20 to 0.18 ± 0.15. Five of 22 patients (22.7%) gained more than 0.2 logMAR, including 1 of 10 patients (10.0%) in the BF group, 2 of 7 patients (28.6%) in the HMD group, and 2 of 5 patients (40.0%) in the BF + HMD group. No significant differences in clinical characteristics were identified among the three groups.

**Conclusions:**

Binocularity-stimulating therapy is somewhat beneficial in children with residual amblyopia and might be attempted when children no longer benefit from sufficient long-term period of occlusion therapy.

## Background

Most common treatments for amblyopia are monocular patching or penalization. By depriving the vision of sound eye, suppression of the amblyopic eye is eliminated and visual experience promote development or recovery of visual acuity of amblyopic eye. However, the response to patching usually reaches a plateau before vision in the amblyopic eye equals that of the sound eye [1–4], a condition referred to as residual amblyopia. Many amblyopes do not achieve a normal visual acuity, regardless of their patching compliance, and amblyopia often recurs after successful treatment in 25–50% of children [5–7]. In addition, older children and adults with amblyopia are rarely treated by conventional treatment.

Recent studies have reported that abnormal binocular interactions play a key role in amblyopia [8–13]. Binocularity-stimulating therapies on amblyopia using perceptual learning or dichoptic stimulus presentation have been introduced [9, 10, 14–16]. The mechanism of dichoptic presentation is presenting a strong stimulus to the amblyopic eye and a weak stimulus to the normal sound eye. Many devices can be used for dichoptic presentation: head-mounted display (HMD) [11], liquid crystal display (LCD) shutter glasses [17], 3-dimensional (3-D) shutter glasses [18, 19], and an iPad [20–23]. Researchers have shown that this type of therapy is effective in treating childhood amblyopia, especially binocular iPad games [20, 23, 24]. However, these studies mostly examined pediatric patients with recent amblyopia diagnoses, and the effect of binocularity-stimulation therapy on children with residual amblyopia (i.e., following patching and/or atropine therapy) has not yet been reported.

We previously developed a new software program which directly targets the binocular function using dichoptic presentation [25]. This program presents 3-D images in a virtual reality environment using a complete split screen view. The visual input to both eyes is controlled using an HMD. We evaluated the effectiveness of binocularity-stimulating treatment in children with residual amblyopia following occlusion therapy for more than 6 months using the HMD and Bangerter foil (BF).

## Methods

### Subjects

A medical chart review was performed on the prospectively collated subjects patients with amblyopia caused by strabismus and/or anisometropia between 2015 and 2016 at Seoul National University Hospital, South Korea. Patients with residual amblyopia following > 6 months occlusion therapy, who had good occlusion therapy compliance, and who underwent binocularity-stimulating treatment were included.

All patients underwent cycloplegic refraction at the first clinic visit to determine refractive error, which was converted to spherical equivalent values for statistical analysis (measured in diopters [D]). Myopia was defined as a negative spherical equivalent and hyperopia was defined as a positive spherical equivalent. Visual acuity was measured using a Snellen visual chart at every visit by one experienced examiner and was converted to the logarithm of the minimum angle of resolution (logMAR) for all data analyses. Ocular alignment was evaluated using the alternate prism cover test with accommodative targets for near (0.33 m) and distance (6 m) fixation. Stereoacuity was tested using the Titmus stereotest (Stereo Optical, Chicago, Illinois, USA). All values were transformed to log arcsec for the purpose of analysis.

Amblyopia was defined as an interocular difference of visual acuity between two eyes at least 0.2 LogMAR (2 lines). Types of amblyopia were divided as follows: anisometropic, strabismic, and combined. Anisometropic amblyopia was defined if there was a difference of at least 1.0D in spherical equivalent or 1.5D in astigmatism between the two eyes with no measurable strabismus. Strabismic amblyopia was defined as amblyopia in the presence of a heterotropia at distance and/or near fixation with a spherical equivalent interocular difference < 1.0D and < 1.5D interocular difference in astigmatism. The deviation is within 8 prism diopters with a history of strabismus surgery or resolution of misalignment after spectacle correction. Combined amblyopia was defined as amblyopia in the presence of both strabismus and anisometropia.

Patients with congenital or acquired ophthalmic conditions (e.g., optic nerve disease, glaucoma, media opacity, or cataract), systemic disease (e.g., neurologic disorders, developmental delays), or poor treatment compliance were excluded.

This study was approved by the Institutional Review Board of Seoul National University Hospital in South Korea and the study protocol followed the tenets of the Declaration of Helsinki. Written informed consent was obtained from the patients’ parents or guardians and patients more than 7 years.

### Binocularity-stimulating treatment

Patients prospectively underwent one of three randomly chosen types of binocularity-stimulating treatment. We used the randomly generated numbers using computer program. One physician who unware of this study generated the program and sealed sequentially numbered envelopes, which were concealed from investigators. After confirming eligibility and obtaining written informed consent, one of us (H-JL or S-JK) opened a sealed envelope, and assigned the patient to the appropriated treatment. These included BF, HMD games, and combination BF/HMD game therapy and binocularity-stimulating treatment was performed for at least 2 months.

**Fig. 1:**
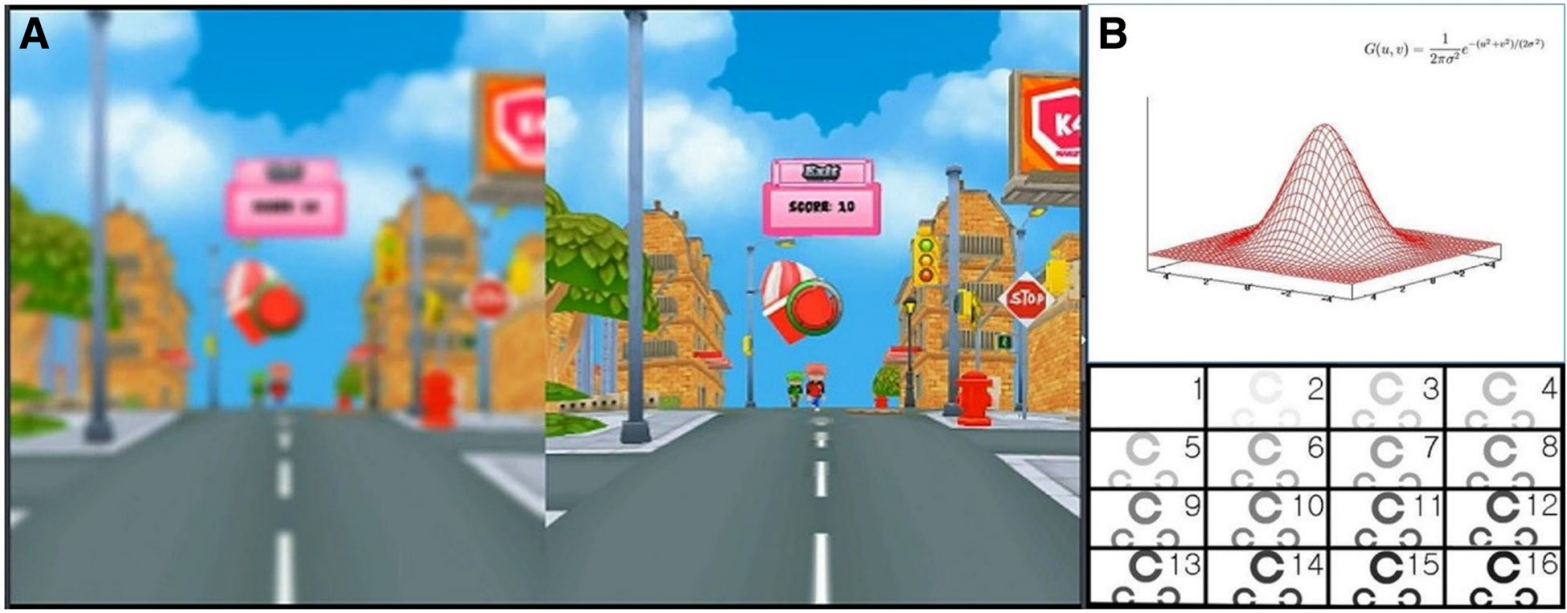
The developed software program named “Ice Cream Truck” game. **a** Example of blur-applied screenshot of the game. It separate the 3D images and control the visual inputs into the both eyes by increasing the contrast and intensity of the 3D target to the amblyopic eye (right) and decreases those to the normal sound eye (left). **b** 16 level of Gaussian blur method applied in this software program

Group 1 underwent BF with 0.6-, 0.4-, and 0.2-strength BF. The BF with a similar level to the amblyopic eye was chosen and the appropriate BF was applied on the glasses of the sound eye for 6 h a day. Group 2 underwent HMD game therapy using the “Ice Cream Truck” game on an HMD for 30 min a day. This game is a casual shooting game that requires players to throw ice cream to kids running towards them. The game is presented on a split screen, which allows independent control of 3-D image contrast and intensity using the 16-level Gaussian blur method. The amblyopic eye is presented images with increased contrast and intensity, while the sound eye is presented images with decreased contrast and intensity (Fig. 1). The player has the ability to select gameplay level as normal, expert, or hard. Group 3 underwent BF/HMD game combination therapy. Patients watched a video or played the 3-D game in a virtual reality environment using HMD for 30 min a day with BF on glasses of the sound eye.

### Assessment of effectiveness of binocularity-stimulating treatment

Patient age, sex, amblyopia type, visual acuity, and treatment duration were investigated. At baseline and at each follow-up visit, best-corrected visual acuity was measured in each eye. After at least 2 months of binocularity-stimulating treatment, baseline and final visual acuity were compared. We also evaluated the number of patients with a vision improvement of 2 Snellen lines (0.2 logMAR) or more after binocularity-stimulating treatment.

### Statistical analyses

The ANOVA test and Fisher’s exact test were performed using SPSS software (Version 16.0 for Windows; SPSS Science, Chicago, IL). For all tests, *P*-values < 0.05 were considered statistically significant. Continuous variables are reported as mean ± standard deviation.

## Results

### Demographic and clinical characteristics of subjects

Of total 22 patients (15 males) included, 10 patients were treated with BF, 7 patients were treated with HMD games, and 5 patients were treated with BF/HMD game combination therapy. The visual acuity at the initial visit was 0.73 ± 0.47 (range 0.2~ 1.8) LogMAR. Seven patients had anisometropic amblyopia, 8 patients had strabismic amblyopia, and 7 patients had combined amblyopia. The mean occlusion therapy duration was 2.5 ± 1.1 years (range: 0.7–4.7 years), and LogMAR visual acuity after occlusion was 0.22 ± 0.20 (range: 0.05–1.0). Occlusion therapy led to a visual gain of at least 0.2 LogMAR in 19 of 22 patients (86.4%). Mean age at the time of binocularity-stimulating treatment was 8.7 ± 1.3 years (range: 6.7–11.1 years) and mean duration of binocularity-stimulating was 4.4 ± 1.8 months (range: 2.1–8.1 months, Table 1).

**Table 1:** Demographic and clinical characteristics in children with residual amblyopia following occlusion for more than 6 months

	Total (*n* = 22)
Sex (male:female)	15:7
Refractive error (diopters)	
Amblyopic eye	+ 3.16 ± 4.10 (range − 5.50~ 7.50)
Fellow eye	+ 2.31 ± 2.67 (range − 2.25~ 7.75)
Laterality of amblyopic eye (right:left)	7:15
Types of amblyopia (A:S:C)	7:8:7
VA at initial visit (LogMAR)	0.73 ± 0.47 (range 0.2~ 1.8)
Duration of occlusion (years)	2.5 ± 1.1 (range 0.7~ 4.7)
VA after occlusion (LogMAR)	0.22 ± 0.20 (range 0.05~ 1.0)
Age at binocular treatment (years)	8.7 ± 1.3 (range 6.7~ 11.1)
Stereoacuities at binocular treatment (Logarcsec)	2.3 ± 0.2 (range 1.9~ 2.6)
Duration of binocular treatment (months)	4.4 ± 1.8 (range 2.1~ 8.1)
VA after binocular treatment (LogMAR)	0.18 ± 0.15 (range 0.0~ 0.5)

Continuous variables are reported as mean ± standard deviation

Abbreviations: A anisometropic, S strabismic, C combined, VA visual acuity, LogMAR logarithm of the minimum angle of resolution

### Effect of binocularity-stimulating treatment on visual acuity

**Fig. 2:**
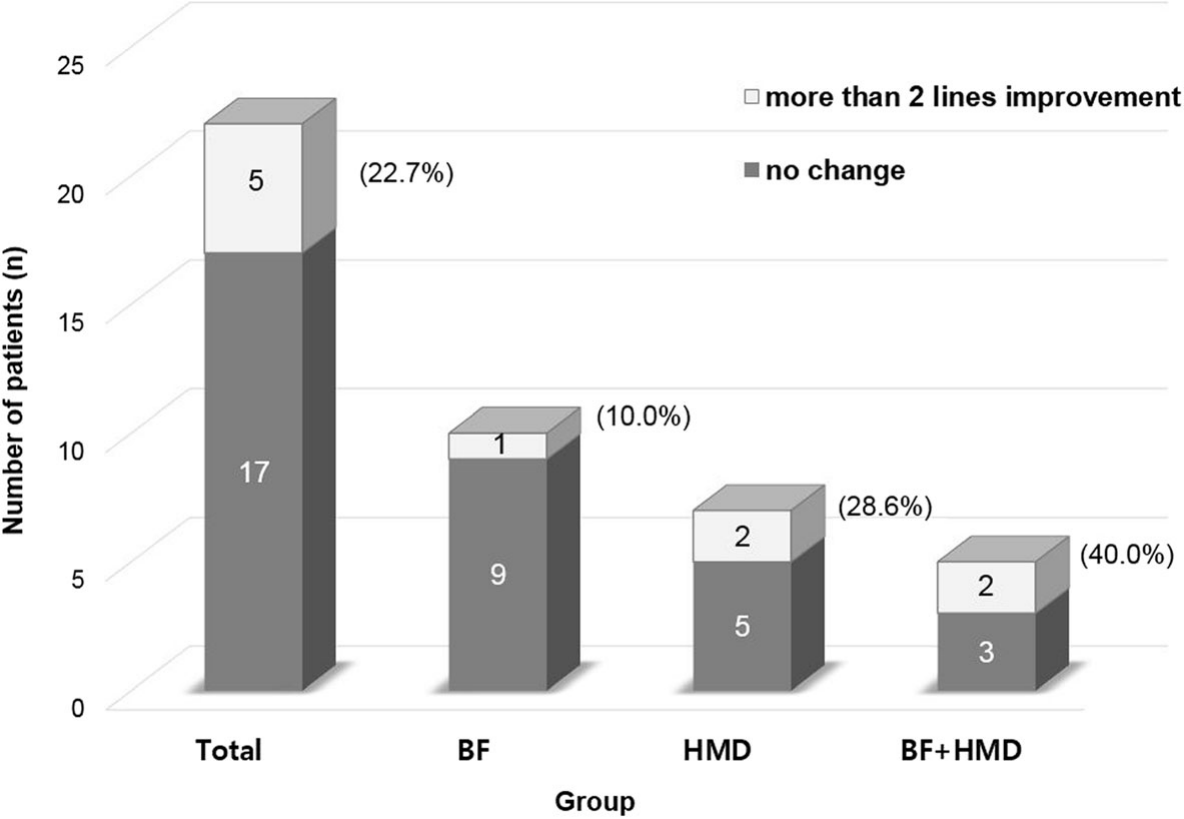
Distribution and change of visual acuity in children with residual amblyopia following occlusion for more than 6 months. Five patients (22.7%) presented more than 0.2 logMAR improvement of visual acuity: 1 of 10 patients (10%) in the BF group, 2 of 7 patients (28.6%) in the HMD group, and 2 of 5 patients (40%) in the BF + HMD group

The visual acuity in amblyopic eye was changed from 0.22 ± 0.20 LogMAR to 0.18 ± 0.15 LogMAR after binocularity-stimulating treatment (*P =* 0.252). Of total 22 patients, there were no significantly different factors including sex, amblyopia type, and binocular treatment age, according to the types of treatment (Table 2). Five patients (22.7%) gained more than 0.2 logMAR of vision (1 of 10 patients [10%] in the BF group, 2 of 7 patients [28.6%] in the HMD group, and 2 of 5 patients [40%] in the BF + HMD group, Fig. 2).

**Table 2:** Comparison of clinical factors and visual acuity in children with residual amblyopia following occlusion for more than 6 months according to the treatment modalities

	BF (*n* = 10)	HMD (*n* = 7)	BF + HMD (*n* = 5)	*P* value
Sex (male:female)	6:4	5:2	4:1	0.852^b^
Types of amblyopia (A:S:C)	3:5:2	3:2:2	1:1:3	0.634^b^
VA at initial visit (LogMAR)	0.66 ± 0.44	0.77 ± 0.53	0.80 ± 0.58	0.844^a^
Duration of occlusion (years)	2.9 ± 1.3	2.6 ± 1.0	1.8 ± 0.5	0.215^a^
VA after occlusion (LogMAR)	0.17 ± 0.12	0.30 ± 0.32	0.21 ± 0.11	0.411^a^
Age at binocular treatment (years)	8.3 ± 1.2	8.7 ± 1.5	9.5 ± 1.4	0.289^a^
Duration of binocular treatment (months)	4.7 ± 2.4	4.2 ± 1.4	4.1 ± 1.1	0.795^a^
VA after binocular treatment (LogMAR)	0.19 ± 0.17	0.17 ± 0.07	0.17 ± 0.19	0.958^a^

Continuous variables are reported as mean ± standard deviation

Abbreviations: BF Bangerter foil, HMD Head-mounted display, A anisometropic, S strabismic, C combined, VA visual acuity, LogMAR logarithm of the minimum angle of resolution

^a^ANOVA test

^b^Fisher’s exact test

## Discussion

Though the mainstay treatment for unilateral amblyopia has traditionally been penalization of the sound eye, there is growing interest in the role of binocular treatments. As further clinical evidence on amblyopia management is accumulated, there may be a shift from penalization and an inclination toward binocular stimulation to improve binocular interaction and promote binocularity.

Binocularity-stimulating treatments include the use of movies and video games displayed on a split screen, with some media components presented in low contrast images for the sound eye and high contrast images for the amblyopic eye. Li et al. [26] examined the effect of watching three dichoptic movies per week for 2 weeks on 8 amblyopic patients (4 patients with anisometropic amblyopia, 1 patient with strabismic amblyopia, and 4 patients with combination amblyopia) that were 4–10 years old. Before treatment, the amblyopic eye visual acuity ranged from 0.24–1.20 LogMAR. They reported that the mean improvement in visual acuity was 2 lines. However, their study only included a small number of patients and had a short follow-up period (2 weeks).

Vedamurthy et al. [27] conducted a larger study on older patients that compared amblyopic patients who watched movies with a patch (*n* = 15 patients) to those who played dichoptic video games (*n* = 23 patients). In each treatment group, approximately half of patients had anisometropic amblyopia and half of patients had strabismic amblyopia. Participants were required to perform their assigned visual activities for 1.5–2 h at least 2–5 times per week for a total of 40 h. They found that the dichoptic video game group had an overall improvement in stereopsis and contrast sensitivity shortly after and 2 months after initiating the intervention.

These prior studies included patients with newly diagnosed amblyopia and treated patients with binocularity-stimulating therapies without any experience of conventional treatment (e.g., patching) [20, 23, 24, 26]. In contrast, our study included amblyopic children who had reached a treatment response plateau after a sufficiently long period of occlusion therapy (residual amblyopes). Residual amblyopia is generally considered to be an untreatable condition, and conventional therapies offer no options for further visual acuity improvements. Before binocular therapy, mean occlusion duration was 2.5 ± 1.1 years and occlusion compliance was good. Even though 86.4% of our patients gained at least 2 lines of vision with more than 6 months of patching, vision in the amblyopic eye had still not reached that of the sound eye. Therefore, different treatments were needed to further improve visual acuity.

We tried binocularity-stimulating treatment using BF and HMD games. Chen et al. [28] reported that BF can immediately reduce suppression and promote binocular summation for mid/low spatial frequencies in observers with amblyopia. In addition, we previously developed a new software program that directly targets binocular function with dichoptic presentation [25]. This program presents 3-D images in a virtual reality environment using an HMD. The system targets binocular function by presenting 3-D images on a split screen. In the virtual reality environment, image contrast and intensity can be independently adjusted for each eye and were increased in the amblyopic eye and decreased in the sound eye. Therefore, patients are forced to use both the sound and amblyopic eye to successfully play games or watch movies. In the present study, mean patient age at the time of binocular treatment was 8.7 ± 1.3 years and mean binocularity-stimulating treatment duration was 4.4 ± 1.8 months. All patients had good therapy compliance. Of the 22 patients included, 5 patients (22.7%) gained more than 0.2 logMAR of vision. Even though we only examined a small number of patients, this result could be meaningful because we only included residual amblyopes.

Our study had several limitations. First, the number of subjects was small and there was no control group which was observed without any treatment. We could not include the control subjects because of ethical issue. In addition, even if we assigned the treatment randomly by masked physician, participants and investigators were not blinded after treatment. To check the compliance and detect any complications/side effects following binocular treatment, we had to know the types of treatment. However, it would be the major limitation, so further blinded prospective research on a larger group of amblyopic children and adults is necessary to prove binocular treatment efficacy. Second, images presented by the HMD had a relatively low resolution. Therefore, display resolution improvements are needed. Investigations to determine optimum utilization in the clinic and at patient homes are also needed.

## Conclusions

To the best of our knowledge, this is the first study to report results of binocularity-stimulating treatment in children with residual amblyopia. There may be some benefit of binocularity-stimulating treatments in residual amblyopic children. Therefore, binocularity-stimulating treatments should be considered in children with residual amblyopia following long-term occlusion therapy.

## Abbreviations

BF: Bangerter foil
D: Diopters
HMD: Head-mounted display
